# The learning curve of retinal detachment surgery

**DOI:** 10.1007/s00417-021-05096-1

**Published:** 2021-02-05

**Authors:** Viola Radeck, Horst Helbig, Teresa Barth, Maria-Andreea Gamulescu, David Maerker, Philipp Prahs

**Affiliations:** Department of Ophthalmology, University Hospital of Regensburg, Franz-Josef-Strauss-Allee 11, DE-93042 Regensburg, Germany

**Keywords:** Buckle surgery, Learning curve, Retinal detachment surgery, Training, Vitrectomy

## Abstract

**Purpose:**

To investigate the learning curve of vitreoretinal (VR) surgeons beginning training in retinal detachment (RD) surgery.

**Methods:**

The files of all consecutive patients undergoing VR surgery for uncomplicated RD between Jan 2005 und Mar 2020 were retrospectively reviewed. Successful outcome was defined as no retinal redetachment within 3 months after surgery.

**Results:**

Ten surgeons started their VR career during this period. Together, these 10 surgeons performed 3786 RD operations (mean 379; median 251; range 71–1053). Primary success rate after one operation was 90% (3420 of 3786). When starting to operate retinal detachments, VR surgeons had a primary success rate of about 80%. Redetachment rates steadily decreased and stabilized at just under 10% after about 200 operations. Beginners needed more than twice the time for the procedure compared to experienced surgeons. The individual learning curves varied widely. In our series, female surgeons seem to have a faster learning curve.

**Conclusion:**

RD surgery performed by VR surgeons in training had acceptable results. With increasing experience, success rates continuously improve reaching stable levels after approximately 200 operations. The training of VR surgeons requires considerable resources.

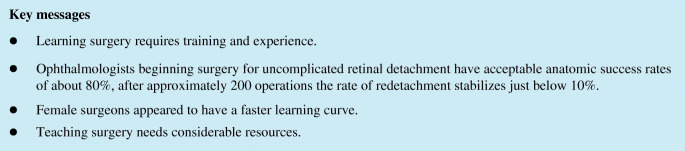

## Introduction

“Doctor, how many times have you performed retinal detachment (RD) surgery?” is a frequently asked question during the conversation before surgery, especially if the surgeon looks young.

Subspecialization and surgical training in ophthalmology are organized differently in different countries. Most healthcare systems provide no financial compensation for teaching surgery. Some have formal fellowships for subspecialties; others have training in general ophthalmic surgery following an ophthalmology residency. In Germany, the specialization in ophthalmology does not require intraocular surgery (except intravitreal injections), and thereafter, no further formal requirements for surgical training exist.

The law of patient rights in Germany (“Patientenrechtegesetz” BGB §630h Abs 4) determines that if a beginner performs surgery and complications occur, the lack of experience and missing know-how of the surgeon is considered responsible as long as the surgeon does not provide evidence to the contrary (reversal of the burden of proof).

RD surgery is a field of ophthalmology, which unlike other subspecialties is not approaching “life style medicine.” Successful surgery means vision; no treatment or failure means blindness to the patients. However, the demands and expectations have grown enormously, and increasingly frequent patients sue their surgeon because of discomfort such as metamorphopsia after successful RD surgery, even with full vision.

This sets the frame and environment for teaching complex surgery like VR surgery for RD.

“Experience matters” appears intuitive for surgical performance. Can this hypothesis be proven and how long does it take for a VR surgeon to learn RD surgery? What is the effort to teach a VR surgeon and what does that mean for the allocation of resources for surgical training? Few studies address this question in RD surgery with different methods and varying results. Here we report the success rates and learning curves of 10 VR surgeons beginning with RD surgery with an average count of close to 400 consecutive operations each. Altogether, nearly 4000 operations were analyzed, the largest series being reported in the literature.

## Materials and methods

Retrospectively, the files of all patients undergoing primary rhegmatogenous RD surgery between January 2005 and March 2020 in our hospital were reviewed. The inclusion criteria were eyes with uncomplicated rhegmatogenous RD undergoing buckle surgery or vitrectomy. Excluded were eyes with PVR grade C or more, eyes with a history of penetrating eye injury, or a history of other vitreoretinal procedures in the past. “Complicated” cases such as eyes with high myopia with macular holes and eyes with retinopathy of prematurity (ROP) and syndromic diseases were also excluded from the analysis.

Failure was defined as diagnosis of redetachment documented in the patient file within 3 months after primary surgery. All patients with surgery between 2007 and 2012 were contacted by mail to detect further redetachments and to get the permission to ask the treating ophthalmologist for the actual status and BCVA. From these 1290 patients, no redetachment was reported, so that we are confident that only few redetachments were missed, who may have had their follow-up in other clinics. Our hospital is the only center for vitreoretinal emergencies in the area serving a population of about 2–3 million people.

Ophthalmology residency programs in Germany take 5 years but do not include intraocular surgery, except intravitreal injections. Training in VR surgery and cataract surgery began in parallel in most cases. General anesthesia is used for all teaching procedures whenever possible. The VR surgical education program initially includes assisting and observing experienced surgeons. Handling under the microscope is then taught with simple extraocular procedures such as conjunctival and scleral sutures or amnion membrane transplantation. Then single steps such as preparing access through the pars plana to the vitreous cavity and cutting vitreous or laser coagulation are performed by the trainee. First complete procedures performed by the trainee under supervision are easy VR cases like removal of liquid silicone or vitreous hemorrhage in pseudophakic eyes. The RD cases included and analyzed in the present study were completely performed by the surgeon in training, and the first procedures were supervised by an experienced surgeon. Thereafter, an experienced surgeon was not present in the OR, but on stand-by for immediate support if required. For these first operations, whenever possible, simple cases were selected (limited detachment, single tear, good view). Allocation of cases to experienced surgeon and trainee had to be done in an individualized and flexible manner, depending on the complexity of the case as well as on the availability and individual experience of each surgeon. Combined cataract and VR operations were performed by the same surgeon in training once sufficient experience with cataract surgery had been acquired typically after approximately 100 phakoemulsifications.

To characterize the background of the trainees at their first RD surgery, data on previous surgical experience was collected from the electronic files and from of the respective surgeons’ records.

The study was approved by the local ethic board and adheres to the principles of the declaration of Helsinki.

## Results

Five thousand one hundred one eyes with uncomplicated primary rhegmatogenous RD were operated between January 2005 and March 2020 by 13 surgeons at the university hospital of Regensburg. Ten surgeons started their VR career during this period. These 10 surgeons performed 3786 RD operations (mean 379; median 251; range 71–1053). Overall, primary success rate after one operation was 90% (3420 of 3786).

In Table 1, the baseline data of the 3786 eyes operated for RD is listed. Table 2 shows the type of surgery being performed. It should be noted that during the observation period from 2005 to 2020, some changes in techniques and concepts occurred. In the beginning, more buckle surgery was performed, and vitrectomy after 2010 was mainly performed with sutureless 23 G or 25 G systems, whereas before 20G, sutured technique was used. At the end of the period, more combined phakovitrectomies were performed. In 1001 eyes, a combined vitrectomy with cataract surgery was performed.

**Table 1:** Baseline data of the 3786 eyes operated for retinal detachment

Age (mean)	61.5 years	
Gender		
Male	2233	59%
Female	1553	41%
Side		
Right eye	1897	50.1%
Left eye	1889	49.9%
Macula status		
Attached	1969	52%
Detached	1817	48%
Lens status		
Phakic	2521	66%
Pseudophakic	1255	33%
Aphakic	10	0,3%

**Table 2:** Types of surgery performed to repair retinal detachment

Vitrectomy gas	2919	77%
Segmental buckle	418	11%
Buckle vitrectomy gas	174	5%
Vitrectomy silicone	148	4%
Encircling buckle	100	3%
Buckle vitrectomy silicone	27	1%
All	3786	100%

**Fig. 1:**
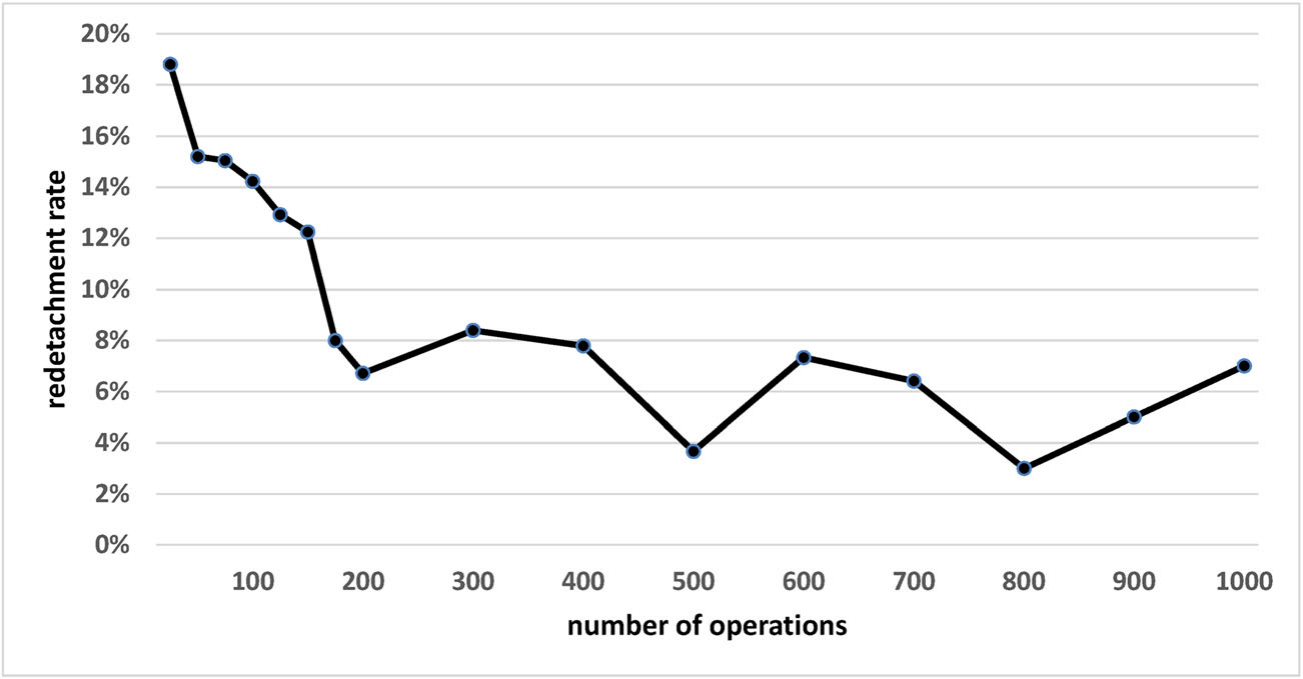
Pooled redetachment rates of 10 VR surgeons in training with increasing experience. The corresponding data are shown in Table 1

**Fig. 2:**
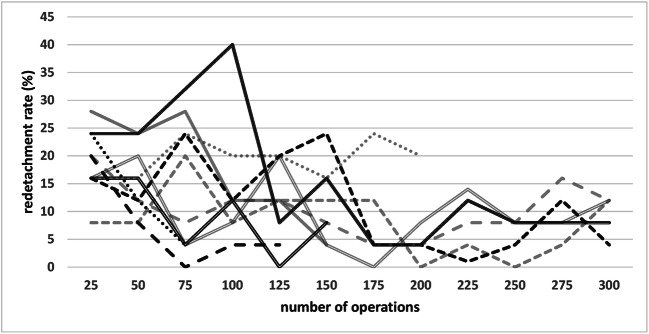
Individual redetachment rates of 10 VR surgeons in training with increasing experience

**Fig. 3:**
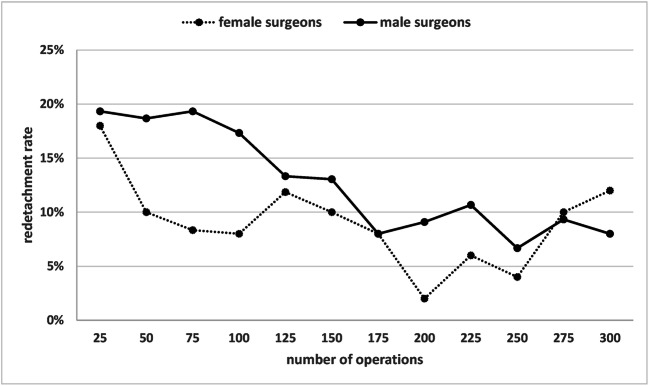
Pooled redetachment rates of RD surgery in 4 female and 6 male VR surgeons in training with increasing experience of the surgeons

Figure 1 shows the pooled redetachment rate of all 10 surgeons with increasing surgical experience. The respective data is shown in Table 3. Figure 2 shows the individual rates of the 10 surgeons with increasing experience. In Fig. 3, the pooled redetachment rates of 6 male and 4 female surgeons are depicted.

**Table 3:** Data for Fig. 1 showing pooled redetachment rates of 10 VR surgeons in training with increasing surgical experience

Surgical experience Number of operations	25	50	75	100	125	150	175	200	300	400	500	600	700	800	900	1000
Number of surgeons	10	10	10	9	9	8	6	6	5	5	3	3	3	1	1	1
Number of operations performed by the surgeons with respective surgical experience	250	250	246	225	209	188	150	149	500	391	300	300	281	100	100	100
% Redetachment	18.8	15.2	15.0	14.2	12.9	12.2	8.0	6.7	8.0	8.0	3.7	7.3	6.4	3.0	5.0	7.0

At the time of the first complete RD surgery analyzed in this study, the trainees were 6.2 ± 1.1 (range 4–8) years in ophthalmology. They had assisted in 112±79 (range 58–300) VR procedures, had performed parts of the surgery in 32±24 (range 6–35) cases, and had completely performed on their own 19±9 (range 6–35) easy VR procedures other than RD cases (e.g., vitreous hemorrhage, removal of liquid silicone). The VR surgeons had performed 27±23 (*n*=9, range 4–71) cataract extractions before their first VR operation for RD; one surgeon had started with cataract surgery earlier and had 354 phakoemulsifications.

From the first 250 RD operations (25 of each of the 10 surgeons in training), in 146, a senior surgeon was supervising the complete procedure. The redetachment rate of the 146 supervised procedures was 18% (27/146), and the redetachment rate of the non-supervised operations was 19% (20/104).

Duration of the procedure was 87 ± 27 min (mean + SD, min 34, max 191, *n*=250) for the first 25 RD operations of all 10 surgeons in training. Average time for surgery for 2 surgeons (last 25 procedures each in 2020) having experience with more than 1000 RD procedures was 39 ± 10 (min 22, max 70, *n*=50) (see also Table 4).

**Table 4:** Cutting-suture time of the first 25 RD operations of all 10 trainee surgeons (1–10) and the time of the procedure of two surgeons with large experience of more than 1000 VR operations for RD (A, B)

Surgeon	1	2	3	4	5	6	7	8	9	10	A	B
Time of procedure, mean of 25 procedures (min)	70	98	93	75	86	75	95	108	87	82	38	40
SD (min)	7	23	26	18	15	15	34	32	31	25	9	12
Minimum time (min)	34	67	55	58	63	47	55	62	51	54	22	22
Maximum time (min)	107	170	172	124	113	103	159	191	170	160	62	70

## Discussion

RD surgery is considered to be a benchmark operation for systematic evaluation of ophthalmic surgery [1]. It provides important data for the quality of surgical performance in ophthalmology, which in many other cases is difficult to measure. Here the outcome is “black” (redetachment) or “white” (retina remains attached). Therefore, we have chosen the attached retina as criterion for success. However, retinal reattachment is not the only factor relevant for the patient in RD surgery. Visual acuity depends on many variables, and since limited postoperative visual acuity is not necessarily due to an unsuccessful surgery, visual acuity was not included in the analysis. Other surgeon-associated factors however, like retinal folds, may be massively disturbing despite reattached retina and good visual acuity. Perfluorcarbon leftovers (subretinal or preretinal) may also cause major symptoms, but were also not analyzed in the present study.

Our results show that beginning surgeons have a primary success rate of about 80% for their first RD operations, which is quite an acceptable rate, showing that it is reasonable to have beginners perform RD surgery. The variability of the individual learning curve is relatively high, as shown in Fig. 2. Many factors, especially case selection, intensity of previous training experience, and individual surgeon factors, may explain these differences. After about 200 operations, the redetachment rate stabilizes just below 10%.

In the literature, varying results have been published from studies using different methods. Our approach, to observe the success rates of a relatively large number of VR surgeons from their beginning, has not been reported so far. Older and smaller series suggested that surgical experience has an effect on RD surgery outcome [2]. Primary vitrectomy for RD has been described to require 120–180 cases or 2–3-year experience until redetachment rates remain stable. On the other hand, even for the first cases in beginners, acceptable success rates were reported [3]. Keller concluded that the first 100 RD operations were crucial when monitoring the learning curve of surgical performance [4]. Sagong suggested that only 30 cases were necessary to reach stable success rates in buckle surgery [5].

On the other hand, Ehrlich et al. more recently found no difference in success rates with small gauge vitrectomy between fellows and consultants [6]. Mazinani et al. also described that primary anatomical success rates did not correlate with the number of vitreoretinal procedures performed by each surgeon [7]. However, a certain improvement of success rates was found in some surgeons, when the first and the second half of the observation period was compared [7]. An accompanying editorial [8] addresses some fundamental questions of learning and teaching surgery. Volume alone does not account for skill levels [9]. The concept of “deliberate practice,” discussing and watching cases of others, especially surgical failures, sharing experience, and discussion amongst colleagues of similar level of experience and with their mentors, can largely improve performance, leading finally to a level of “excellence.” Teaching on the other hand is a good opportunity for senior surgeons to further improve performance, as young fellows challenge fixed dogmas and thus may prevent their mentors from becoming rigid in their long-standing habits.

Interestingly, Heimann [10] described that younger, less experienced surgeons may have even better results than more experienced ones. He discussed case selection as a possible reason but also mentioned that younger surgeons might be more open to adapt to technical and conceptual advancements, whereas older surgeons may fall behind. The surgeon factor is a delicate item, burdened with personal vanities and difficult to discuss in the scientific literature [11].

There has been a great deal of discussion with respect to a recent study showing that patients operated by female surgeons had a lower mortality rate [12]. It was speculated that the gender barriers women experience during their surgical training and daily routine might lead to a selection of a cohort of female surgeons that are proportionally more skilled, motivated, and harder working. In our series, too, it appeared that female surgeons might have a faster learning curve than male surgeons. However, other factors like case selection, publication bias, or coincidence must be considered possible explanations, which prevents definite conclusions.

Overall, the primary success rate of 90% was relatively high in our study, considering the skill levels of beginning surgeons. This may be explained by the exclusion of complicated cases. The UK database found an 87% success rate [13] and a recent Canadians study 85% [14], and the ERVS found 85% [15]. With specialized VR surgeons, the standard for primary success was set in the region of 85–90% [16]. Furthermore, success rates are highly dependent on case selection. Trainees should whenever possible start with simple cases. Success rates in more difficult cases are likely to improve much later. Even when success rates as demonstrated here reach a stable level, success rates in complicated, unusual cases may improve later on, no longer easily detectable by simple statistical approaches.

The German healthcare system—like many others—has no formal fellowships for subspecialization in ophthalmology. Therefore, no specific education program exists. Surgical training usually starts after the residency. Most ophthalmic surgeons in smaller departments have to cover both, anterior and posterior intraocular surgery. Although teaching hospitals, which are mainly under public sponsorship, try to train surgeons in an organized and responsible manner, the specific circumstances require a lot of improvisation and flexibility, especially for unplannable operations such as RD surgery. Experienced surgeons, willing to teach, are the cornerstone for a successful program. Their availability is limited. The term of notice for civil servant physicians is 1 day. In addition, it is mostly not allowed in Germany for pregnant female physicians to perform surgery, and all female surgeons in this study had at least a 1-year baby break. Especially in smaller ophthalmology departments, these uncertainties of planning may be difficult to harmonize and bring in line with patient care, economic constraints, and teaching.

Teaching surgery requires time and resources. We have shown here that trainees need more than twice as long time for a RD operation than experienced surgeons do. Moreover, for the first operations, an experienced surgeon has to be present to safely guide the beginner through the various steps. The effort for educating surgeons is high, and it is mandatory that a healthcare system provides the necessary financial and personnel resources.

Limitations of our study are its retrospective design, a possible selection bias of easier cases for VR surgeons in training and the incomplete follow-up of some surgeons (4 out of 10) that did not accomplish at least 200 RD operations. Prospective studies in general had a tendency to show less positive results compared to retrospective analysis [17, 18]. On the other hand, the 23/25 G arm of the prospective VIPER study also showed a 90% primary success rate [17]. Due to the retrospective design and not complete follow-up of all cases, we acknowledge that we may have missed some failures. Moreover, if the retinal was flat under liquid silicone tamponade after 3 months, it was considered non-redetached, although some redetachments may occur later after removal of the liquid silicone. Other studies used a 6-month period postoperatively before assessing the success of surgery, but the vast majority of redetachments typically occur in the first 3 months [17]. This however should have the same effect on all subgroups. Despite these limitations, our series with nearly 4000 operations performed by 10 VR surgeons provides a profound basis for evaluating the learning curve in RD surgery.

In conclusion, our study shows that VR surgeons in training had a primary success rate of approximately 80% when starting RD operations. The redetachment rate steadily decreased and stabilized just under 10% after about 200 operations. The individual learning curves varied widely. In our cohort, female surgeons in training had a faster learning curve. The training of VR surgeons requires considerable resources.
